# Truth, Racial Healing, and Transformation: Creating Public Sentiment

**DOI:** 10.1089/heq.2021.29008.ncl

**Published:** 2021-09-21

**Authors:** Gail C. Christopher

**Affiliations:** Executive Director, National Collaborative for Health Equity, Washington, District of Columbia, USA.

Abraham Lincoln understood the importance of public sentiment to our democracy when he debated Stephen Douglas in 1858. “In this and like communities, public sentiment is everything,” Lincoln noted. “With public sentiment, nothing can fail. Without it, nothing can succeed.”^1^

A little over a century later, in 1967, President Lyndon Baines Johnson formed the National Advisory Commission on Civil Disorder, headed by Illinois Governor Otto Kerner, Jr. to search for and identify the root causes of the riots that erupted in over 150 cities across the United States during the summer of 1967. When it issued its report the Kerner Commission cited institutional racism, police brutality, and lack of employment opportunity as the causes of the 1967 uprisings. Forty-nine years later, the conclusions cited by the Kerner Commission are still relevant. Institutional and structural racism still perpetuate barriers to employment. Police brutality along with myriad inequities in opportunity for health, housing, education, transportation, and most of the social determinants of wellbeing still persist today. These barriers remain despite significant efforts to close these opportunity gaps and despite the Civil Rights legislative and judicial victories of that era.

The Truth, Racial Healing, and Transformation (TRHT, http://healourcommunities.org) work launched by the W.K. Kellogg Foundation (WKKF) is a national truth and reconciliation process for the United States of America, its territories, and the sovereign nations of indigenous people within its borders. The TRHT, while informed by the well-recognized truth and reconciliation commission (TRC) model, is a unique process designed to reflect, embrace, and address the unprecedented diversity and unparalleled racialized history of the United States. Lincoln knew the primacy of the public “sentiment” in 1858. The Kerner Commission emphasized its importance again in 1968. Its final report, which still stands as the country's most thorough public research on racism put it this way:

The need is not so much for the government to design new programs as it is for the nation to generate new will. Private enterprise, labor unions, churches, foundations, universities, and all our urban institutions must deepen their involvement in the life of the city and their commitment to its renewal and welfare.^2^

In times of palpable and, yes, violent racial strife, civic leaders have understood the primacy of public sentiment and public will in efforts to restore and sustain peace and civility. Yet we have never as a nation implemented a concerted or comprehensive strategy to generate public sentiment and will for a united and healed national consciousness on issues of racial division and inequity. Our centuries-old failure to do so has spawned a legacy of division which today, as in the past, threatens the stability, economic viability, and future of America. TRC is an internationally recognized process of helping divided countries to come back together or “reconcile” after war, human rights atrocities, and sometimes after protracted traumatic divisions. These truth-telling efforts have been used most often to reunite previously warring factions, the most widely recognized of which is the South African TRC process. The process has been implemented over 44 times around the world. The most recent process in Canada, focused on historic mistreatment of indigenous children and forced separation of families, released its report in the Fall of 2015.^3^

TRHT work in the United States leverages the brand and overriding intention of truth and reconciliation models. This U.S. adaptation emphasizes transformation rather than reconciliation. The original TRC model for bringing a country back together through reconciliation is not appropriate in the United States, where racism and the belief in a hierarchy of human value are integral to the nation's foundational processes. For example, Article 1, Section 2, Paragraph 3, said that enslaved persons were to be counted as 3/5 of a whole person. Thus, the TRHT work must be designed to transform the undergirding ideology of a hierarchy of human value and to transform the societal structures that are still supporting this antiquated belief in the lack of inherent worth or value of people of color. Participating TRHT communities will apply a specific TRHT framework in their efforts to reveal the truth and use it to foster healing that can usher in needed public sentiment, indeed political will, to do what has previously been impossible: transform America into a nation that values all people equally and reconstructs itself on that fundamental truth, as aspired to in the Declaration of Independence (U.S., 1776):

All… are created equal and endowed by their Creator with certain unalienable rights, that among these are life, liberty and the pursuit of happiness.

## What Is Public Sentiment?

The Merriam-Webster Dictionary defines public sentiment as follows: “An attitude, thought, or judgment colored or prompted by feeling or emotion.” The Cambridge English Dictionary introduces the word “idea” into its definition of sentiment. “A thought, opinion, or idea based on a feeling about a situation; a way of thinking.” It is the latter, the way of thinking, that has the most relevance to TRHT.

Public sentiment in the 21st century is influenced and assessed in ways that were unimaginable in the 19th and 20th centuries. Information technology capabilities, polling methodologies, communications, media, particularly social media and global influences combine today to create an overwhelmingly dynamic landscape of public opinion. Even so, the issue of racism is no less salient today than it was when Lincoln and Douglas debated, or when President Johnson created the Kerner Commission. Add to the crowded public opinion/sentiment landscape the recent deliberative and participatory democracy movement that has been gaining momentum since the 1990s. People across America in their local communities are engaging on issues of concern and using their muscle to influence policies. As exciting as these citizen engagement developments are, they are not designed to consider conscious or unconscious racial biases. During the early 1990s, I served as the director of the Innovations in American Government Awards Program at Harvard's John F. Kennedy School of Government. Our review teams were always impressed by the emerging participatory governance models such as citizen-based budgeting and deliberative democracy initiatives. Engaging diverse groups of citizens in decision-making and thoughtful deliberation on critical issues seemed to be a good strategy for pushing back against the then (as now) rising tide of anti-government sentiment. Berkeley Professor Ian Haney Lopez's 2015 groundbreaking book *Dog Whistle Politics: How Coded Racial Appeals Have Reinvented Racism and Wrecked the Middle Class* uncovered the solid connection between anti-government sentiment and America's racialized politics, both past and present.^4^ The book provides a platform for a deeper consideration of how economic, legal, and social policy debates can mask deep-seated, but easily manipulated racial biases. The 2016 presidential election did not use coded racist messages but was more overt and directly inflammatory and divisive, seemingly intentionally leveraging racial anxieties and fears to mobilize voters. The election outcome ushered in documented increases in hate crimes, child bullying, and immigrant intimidation along with white nationalist discourse, deemed unacceptable in recent decades.

Lincoln's talk about the power of public sentiment in 1858 and the Kerner commission's call for the public will to engage in urban issues were, I believe, thinly veiled calls for changing hearts and minds about race and racism. The promise of democracy in America cannot be fully realized without responding to this call. WKKF and, to date, over 50 other foundations agree that it is now time for communities throughout America to begin this deliberative racial healing work. We are supporting TRHT coalition efforts locally and nationally that are explicitly and transparently focused on eliminating the very notion, belief, and false ideology of a human value hierarchy based on physical characteristics—racism.

The goal of the TRHT is to change the way the people in America think about issues of racism by exposing and addressing the fallacy of the dominant belief and subsequent societal systems of racial hierarchy. Belief in a hierarchical taxonomy or classification system for the human family was formalized by Swedish botanist Linnaeus in the 1700s. He was building on a then centuries-old Doctrine of Discovery in the Papal Bull issued by Pope Alexander VI on May 4, 1493, which became the basis for all land acquisition by non-indigenous people in the New World. Linnaeus codified a biological system of superiority and inferiority for diverse humans from different geographic locations into “races.” These racial groups also had varying physical characteristics. Behavioral and character traits were assigned to these groups which became the basis of so-called scientific racism. It was used to justify and rationalize violations of humanity for centuries. Although now discredited by science, the legacy of this widely held and deeply embedded belief in racial hierarchy still lives today. It still resides in all of the normalizing, socializing, and standardizing systems and institutions of today. From education to art, to media and religion, to politics and finance, the legacy of racial hierarchy—as a core societal construct—still exists. Public sentiment today in America is still largely shaped by racism.

## The Urgency of Now

Population demographics create an urgency for ridding our society of the burden of racism. According to the U.S. Census data, most of the children born in America today are children of color. The majority of these children are growing up in low-income or impoverished families and communities.^5^ Researcher Dolores Acevedo Garcia calls this double jeopardy, emphasizing the cumulative harms associated with concentrated poverty and racially segregated communities.^6^ While changing demographics create an imperative for eliminating racism on the one hand, fear of increasing diversity has fueled greater resistance on the other.

I have had the privilege of working for the WKKF for the last decade and as vice president for program strategy guiding multiple program areas, including designing and leading the foundation's racial equity and racial healing funding approach. Will Keith Kellogg created the foundation over 80 years ago giving it one simple mandate: the money was to be used to improve the lives of vulnerable children. When the WKKF board members faced the demographic realities of vulnerable children in 2007, they were easily convinced that just dealing with the consequences of racism would no longer suffice. Board members realized that persistent health disparities such as infant mortality, widening wealth divides, and seemingly intractable educational achievement gaps are actually the consequences of structured racism. The foundation's leadership made a bold commitment to becoming the most effective anti-racist organization that promotes racial equity. Although board members, staff, and the CEO have changed during the last decade, WKKF's firm commitment to racial healing and racial equity has remained intact.

In a society where frank discussions about race and racism are all too rare, the Foundation openly advocates for putting racism behind us and acknowledging the damage it has caused. This advocacy enables opportunities for healing and progress, bringing hope for future generations.^7^

The unanimous decision by the board to support the TRHT is further affirmation of its abiding commitment.

Researchers Sean McElwee of Demos and Jason McDaniel of San Francisco State University explain how racial resentment played a larger role in the 2016 presidential election than the popularly touted economic concerns.^8^ Their analysis of recent survey data reveals that opinions about how increasing racial diversity will affect American society had more impact on support for the Republican presidential candidate in the 2016 campaign when compared to support for Republican candidates in the previous two presidential elections. They conclude that the winning presidential candidate successfully leveraged existing resentment toward African Americans in combination with emerging fears of increased racial diversity in America to literally reshape the presidential electorate. Finally, McElwee and McDaniel assert that racial and identity attitudes have further displaced class as the central battleground of American politics.

This, ironically, comes at a time when polling data showed that for the first time in our national history, whites and people of color were moving toward greater consensus about the need to effectively address race relations in this country. In a national poll conducted in 2015, 53%of whites said that more changes are needed to give blacks equal rights with whites, up from just 39%a year earlier.^9^ So, we have the tension between two expanding pools of public sentiment. One is clinging to the mythology of a hierarchy of human value (racism). The other, an increasing segment of the majority population (people in virtually every community) wants to eradicate racism and create equity.

## A Coalition Is Formed

After reviewing the literature on the 44 TRC's around the world, a core guiding principle emerged for creating a successful TRHT for America. It would have to be multisectoral. It's noteworthy to remember that it was also a multisectoral approach that was called for by the Kerner Commission in 1968, but never realized. While in the early months of our work on TRHT, we reached out to and received calls from over 100 diverse organizations that expressed the desire to get involved and help in this unprecedented effort—public, private, nonprofit, faith, academic and research, media, grassroots, advocacy, civil and human rights, and judicial organizations reflect this multisectoral emphasis.

We had already learned from almost a decade of funding the racial equity and racial healing work that the approach had to be racially and ethnically expansive in nature. By focusing on uprooting the absurd belief in racial hierarchy itself, it became quite natural to address the multiple effects and harm caused to many, if not all, population groups. Creating a broad, expansive, and inclusive coalition engaging Native and indigenous groups, Asian American, Pacific Islander, immigrant groups, Latino and Hispanic, African American, Arab American, and European or white Americans is a second core principle of the TRHT work.

## A Vision Guides the Design Process

One of the first steps we asked ourselves was how the belief in a hierarchy of human value became foundational in the United States and how it has been sustained for centuries. We knew that the national narrative was key to embedding and sustaining the belief—and so we were clear that we needed to work on narrative change. There's been an absence of acknowledgement of the harm that has and continues to come from the belief, resulting in the absence of a concerted effort to heal from it—and so we knew that we needed to do work on racial healing. We also saw that the belief has been embedded in our systems—in the ways communities are kept separate from one another, in the laws (the civil and criminal laws and policies that come from them) and in the economy. We set up five design teams to think about how to develop TRHT around these five areas: narrative change, racial healing, separation, the law, and the economy:

- The Narrative Change Design Team examined how to create a more complete and accurate narrative that will help people understand how racial hierarchy has been embedded in our society from the beginning. The team was committed to utilizing all available vehicles to ensure that a more complete and accurate narrative emerges.
- The Racial Healing and Relationship Building Design Team focused on ways all of us can heal from the wounds of the past and build mutually respectful relationships across racial and ethnic lines, relationships that honor and value each person's humanity. The team also explored ways to inform public policies so that they better reflect our common humanity.
- The Separation Design Team examined and found ways to address segregation, colonization, and concentrated poverty in neighborhoods.
- The Law Design Team reviewed discriminatory civil, criminal, and public policies and recommended solutions that will produce a more just application of law.
- The Economy Design Team studied structured inequality and barriers to economic opportunities and developed solutions that will create a more equitable society.

Participants in the design teams worked collaboratively between June and November 2016 to co-design the TRHT approach. The design teams were extremely busy people spread throughout the country working collaboratively, so we needed to develop a creative approach for interaction. Each team of 30–40 people had at least one face-to-face meeting and continued to work through virtual meetings and through an online community.

Unlike many approaches to social change that start from the problem, what's been missing has been a clear vision of a redesigned society that is no longer built on the premise of a hierarchy of human value. Each team discussed and wrestled with how to transform our country by answering the following questions:

- Question 1: What will the country look and feel like when we jettisoned the belief in a hierarchy of human value and the narratives that reinforce that belief?
- Question 2: Where are we now and how did we get here?
- Question 3: What are the key leverage points for jettisoning the belief in a hierarchy of human value?
- Question 4: Who must be involved in order to make the deep and lasting changes we need to make?
- Question 5: What are the key initial activities that need to happen in order to heal from and transform the narrative?

Here are examples of answers to Question 1 from two of the design teams (the Narrative Change and Racial Healing Design Teams).

*Narrative Change*: To achieve “TRHT,” we need strong and accessible narratives that advance the values of fairness, compassion, inclusion, and justice. We need narratives that not only effectively challenge racial hierarchies and discrimination based on white hegemony, but that also encourage new ways to coexist in multiracial communities as a nation. We envision stories being told that are complete and accurate in their portrayals of people of color, who have been overwhelmingly misrepresented, objectified, and stereotyped in U.S. media and cultural institutions since before the nation's founding. As a result, the nation's history and identity have set and reinforced the ideology of false ideology of racial year after year for five centuries, often by making invisible the profound role racial exploitation has played in establishing the nation we now live in.

Changing such long-held ideas won't be easy, but those of us committed to a nation in which we tell complex truths rather than simple lies must take on this work. If we successfully pursue the following analysis and recommendations, we will create a culture populated with diverse positive images of ourselves and each other in our full humanity and potential in all media and cultural settings. Such settings would include literature, museum exhibits, parks, places of worship, schools, magazines, newspapers, music, art, theater, television shows, movies, radio programs, games, and social media. The narrative will foster empathy and connections that allow us to see ourselves in each other and thereby help to eliminate the emotional separation between communities. Americans would overwhelmingly have a full, complex understanding of each other. The infrastructure needed to create and distribute new narratives will be strong, with people of color having plenty of access to the means of production and distribution. Financing for story making and storytelling will be widely available—not controlled by a select few. This would lead to a country living up to its creed, finally realizing its own ideal of freedom.

*Racial Healing*: We imagine an America where all people are seen through the lens of our common humanity and we see ourselves in one another. This new society is characterized by love, interconnectedness, mutual respect, accountability, empathy, honoring nature, and care for the environment. In this society, healing and justice flow from authentic relationships.

Our children and grandchildren feel safe and secure in who they are and proud of their heritage and culture. They are able to look within themselves and to their communities to find their identity; they recognize and value the differences inherent in all of us, while celebrating the common threads that bind us together.

Schools are well funded and recognize that all children have a sacred gift and purpose; they offer early education in the child's own language. Libraries and museums reflect the rich heritage and stories of every group, told from the perspective of each group. Memorials serve as a reminder of suffering but also the effort and strength that emerged from it. We no longer carry the pain, fear, and shame of history, for we have discovered how to look at our past with courage and honesty. Places of worship celebrate diversity and work actively to cultivate equitable empowering life in their communities. Parks and public spaces are accessible to all. Our financial system focuses on supporting the health and welfare of communities. In responding to those who do us harm, our justice system reflects a focus on restorative rather than punitive justice. In jettisoning the belief in a hierarchy of human value based on race, we have also eliminated a hierarchy based on class, gender, age, religion, sexual orientation, and ability. In achieving this vision, we have truly achieved transformation.^10^

## Local Communities and Foundations

### Embrace TRHT

In December, 2016, we convened a national summit in Carlsbad, California (a summary video of which can be seen here: https://youtu.be/xwtnanuRrrc), bringing together people who had been involved in the Design Teams, WKKF grantees, and those who were interested in implementing TRHT in their communities. This was an expansive group of over 570 people from across the United States Native American, Alaska Native, Native Hawaiian, Asian American Pacific Islander, Latino, African American, Arab American, and White from multiple sectors (people from the nonprofit, grassroots, education and academia, business, faith, music and arts, entertainment, journalism, government, justice, youth-serving and philanthropy sectors—including the national racial justice organizations) to fully engage for four full days in racial healing, sharing the incredible results of the national design team process and planning a way forward.

One of the outcomes of the summit was a call for action for an annual National Day of Racial Healing on the day after the Martin Luther King Holiday. On January 17, 2017 (only 5 weeks after the summit where this was recommended), tens of thousands of people in communities and organizations across this country stood together for love on the inaugural National Day of Racial Healing in America. The states of Michigan and Georgia, along with 15 localities issued proclamations to recognize January 17 as the National Day of Racial Healing. For this first annual National Day of Racial Healing, we reached 1,527,228 people on Facebook and had 48,700 impressions on Twitter. The National Day of Racial Healing in 2018 will be on January 16, 2018, the day after the Martin Luther King Day Celebration.

In the Spring of 2017, the WKKF announced funding for the first 14 places implementing the TRHT. In each community, the core group of participants includes philanthropic representatives; elected officials; faith community representatives; grassroots activists; healing practitioners; young leaders; community media/narrative change agents (e.g., the publisher of the local newspaper, head of the local TV station, local bloggers, filmmakers, historians, storytellers, artists); people focused on housing, segregation, and colonization locally; people who work with civil or criminal law or public policy and people who work with changing the local economy.

TRHT participating communities are using the same design process, answering the same key questions but tailoring the recommendations to the needs of their local communities. They are organizing local TRHT coalitions to heal racial wounds of the past, while addressing barriers to success for all people, especially children and people of color. The community-based effort is working with people of all races, ethnicities, and national origins to bring together public officials, community, and civic leaders, churches, schools, foundations, and organizations to implement a TRHT process that engages the community in telling the authentic stories and history of that place, helping people heal the racial divides that keep them apart, and addressing the structural challenges related to segregation and colonization, the law and the economy.

Through the TRHT, participants are developing new skills and capacities at individual, organizational, and community levels to identify biases, behaviors, and institutional practices that perpetuate racial discrimination and divisions, so they can truly begin to transform public sentiments and take actions that lead to new policies and structures in their communities.

In April 2017 in Chicago, 200 people gathered at the Chicago Theological Seminary for a day of racial healing, in which people from all walks of life shared their perspective on racism that they have encountered or even projected onto others. These healing circles are critical to building trust that allows diverse audiences to have genuine conversations about racism and differences, and move forward to address its consequences in their communities.

The work in the 14 places includes comprehensive research of the region's racial past, and an acknowledgement of that history whether it includes lynching or abolitionists. One coalition's participants vow to explore their personal narratives about others; challenge perceptions about race, race relations and racial equity; end personal roles in perpetuating racial division; elevate views of all individuals; and move forward on a path of justice, dignity, and humanity.

These communities are boldly moving beyond dealing with the consequences of racism, which has been the focus of most social justice and advocacy efforts since the 1960s. Now, we are addressing the core fundamental belief itself, the racial hierarchy that fuels racism and the notion that the color of one's skin should dictate their station in life.

## How Can You Get Involved?

The overriding purpose of the TRHT is to improve our capacity as communities and as a country to see ourselves in each other, so that we can shape a more equitable future with opportunities for every child to thrive.

If you share this vision and want to become more involved in the TRHT process, you can do so as an individual, as part of an organization or as a community. More detailed guidance is available for engaging at any of these levels in the comprehensive *TRHT Implementation Guidebook* at www.racialequityresourceguide.org/guides-workshops/trht-implementation-guidebook.^11^

You may want to implement some or all of the TRHT framework within your organization—at least taking on narrative change and racial healing—and participate in the annual National Day of Racial Healing.

## Conclusion

Chicago and Alaska are two of many local U.S. communities that began implementing the TRHT framework in their jurisdictions in the summer of 2017. Each has created a broad multisector coalition that represents diverse racial, ethnic, religious, generational, class, and identity groups. They understand that through engaging and experiencing all their diverse stories and narratives, healing insights are gleaned; and a path toward affirming their shared, common humanity emerges. With funding from WKKF and many other local foundations, all participating communities are now part of an expanding national network of communities, organizations, and individuals working to heal America's unnecessary racial divides. They are all holding themselves accountable for building trust across racial lines, while recognizing and changing limiting and biasing narratives. They have agreed to measure their progress in making sustainable changes that overcome negative consequences of colonization, forced segregation, and concentrated poverty. They are boldly working to transform legal and economic systemic inequities by facing historic and contemporary truths about the role of racism, while finding ways to work together for redress. They are proclaiming a new narrative and creating a new reality that refutes the false ideology of a hierarchy of human value, replacing it with affirmation of our equal humanity and sacred interconnected reality. They are not naive nor are they bitter. They are acknowledging and facing the deep, longstanding racial divisions and biased sentiments in their communities, sectors, organizations, and in their own hearts. But they know these divides are needless and harmful. TRHT participants are leveraging this knowledge to begin the healing journey for the sake of our children, ourselves, and our democracy.

## Notes

***Gail Christopher,*** *former W.K. Kellogg Foundation senior advisor and vice president, is the architect of the TRHT. She is a Fellow of the National Academy of Public Administration and Chair of the Board of the Trust for America's Health.*
